# Trends and determinants of pneumococcal vaccine uptake among U.S. adults, 2019–2024: a pooled cross-sectional analysis

**DOI:** 10.1186/s12889-026-27617-5

**Published:** 2026-05-08

**Authors:** Mary Isha Koroma, Nirajan Budhathoki, Joseph Inungu, Jayalakshmi Gurrala, Victor Ogbonna Okike, Deborah Testimony Cyprian, Ramaprabha Makireddy, Plamedi Ngankusu Manwana, Hina Kainat

**Affiliations:** 1 Master of Public Health Program, School of Health Sciences, Central Michigan University, Mount Pleasant, Michigan, 48858 USA; 2 Department of Public Health Sciences, Henry Ford Health, Detroit, MI USA; 3 Department of professional programs in Human services, East Central University, Ada, OK USA; 4 College of Basic Medical Sciences, Jilin University, Changchun, Jilin China

**Keywords:** Pneumococcal vaccines, Vaccination coverage, Healthcare disparities, Immunization, NHIS, *Streptococcus pneumoniae*, Preventive Medicine

## Abstract

**Background:**

Pneumococcal disease causes substantial morbidity and mortality among U.S. adults, with an estimated 20,000 deaths and 150,000 hospitalizations annually. Despite effective vaccines, coverage has remained below national targets. Pandemic-related disruptions and the 2022 Advisory Committee on Immunization Practices transition to simplified 15/20 valent Pneumococcal Conjugate Vaccine recommendations may have influenced uptake, but their impact has not been fully assessed. To address this gap, this study examines trends and determinants of pneumococcal vaccine uptake among U.S. adults following the pandemic and 2022 guideline changes.

**Methods:**

We conducted a pooled cross-sectional analysis of National Health Interview Survey data from 2019 to 2024, including 166,948 adults aged ≥ 18 years with complete data. Weighted bivariate analyses assessed associations between vaccination uptake and demographic (age, sex, race/ethnicity), socioeconomic (education, income, citizenship), health-related (chronic conditions including Chronic Obstructive Pulmonary Disease, diabetes, asthma, and cancer; smoking; influenza vaccination), and healthcare access factors (insurance, usual source of care, recent provider visit). Multivariable logistic regression identified independent predictors of vaccination. All analyses incorporated National Health Interview Survey complex survey design and sampling weights.

**Results:**

Overall weighted pneumococcal vaccination coverage remained low, fluctuating between 29.6% and 33.2% across survey years. Coverage was highest among adults aged ≥ 65 years (65.7%) but remained substantially below the Healthy People 2020 goal of 90%. Only 23.0% of adults aged 55–64 years reported vaccination despite risk-based recommendations. Disparities persisted across racial/ethnic groups, with Hispanic adults showing the lowest uptake (13.9%) compared to non-Hispanic White adults (29.1%). In adjusted analyses, age ≥ 65 years (odds ratio [OR] = 9.39; 95% confidence interval [CI]: 8.84–9.96), chronic conditions including Chronic Obstructive Pulmonary Disease (OR = 2.04; 95% CI: 1.90–2.20) and diabetes (OR = 1.84; 95% CI: 1.74–1.95), recent healthcare visits (OR = 1.41; 95% CI: 1.31–1.51), and influenza vaccination were strong independent predictors of uptake. Lack of insurance (OR = 0.89; 95% CI: 0.81–0.98), absence of a usual source of care (OR = 0.81; 95% CI: 0.74–0.88), and non-receipt of influenza vaccine (OR = 0.29; 95% CI: 0.28–0.30) were associated with lower odds of vaccination.

**Conclusions:**

Pneumococcal vaccination coverage among U.S. adults remained stagnant from 2019 to 2024, with persistent gaps among younger high-risk adults and racial/ethnic minorities. Healthcare engagement, insurance, provider contact, and influenza vaccination, were strongly associated with uptake. These findings suggest that risk-based screening, provider recommendations, and improved access for underserved populations may help address coverage gaps.

## Introduction

*Streptococcus pneumoniae* (pneumococcus) is a gram-positive bacterium with more than 100 known serotypes and is transmitted via respiratory droplets. It is a leading cause of serious illness, including sepsis, meningitis, and pneumonia, among children and adults worldwide [1, 2]. In 2021, pneumococcal disease was estimated to cause about 505,000 deaths globally, with young children and older adults at highest risk [3]. In the United States, pneumococcal infections among adults lead to substantial morbidity and an estimated 20,000 deaths annually, and pneumococcal pneumonia accounts for approximately 150,000 hospitalizations each year [4, 5]. In reality, these figures likely underestimate the true burden, as many cases go undiagnosed in a healthcare system without universal coverage.

Pediatric pneumococcal conjugate vaccine programs, introduced in 2000, have reduced disease incidence across all age groups through indirect (herd) protection [6, 7]. However, serotype replacement has partially offset these gains, and older adults and those with chronic conditions remain at elevated risk [8, 9]. For these groups, direct vaccination remains the primary prevention strategy. The Advisory Committee on Immunization Practices (ACIP) recommends pneumococcal vaccination for all adults aged 65 years or older and for adults aged 19–64 years with selected chronic or immunocompromising conditions [10]. The period of 2019–2024 represents a critical evolution in these strategies. During this time, the adult immunization schedule shifted from complex multi-dose series involving 13-valent (PCV13) and 23-valent (PPSV23) vaccines toward simplified regimens using newly approved higher-valency conjugate vaccines (PCV15 and PCV20) [11]. ACIP recommended either PCV20 alone or PCV15 followed by PPSV23, simplifying dosing and expanding serotype coverage [11].

Despite long-standing recommendations and national targets, adult pneumococcal vaccination coverage remained below desired levels prior to the period examined in this study. Healthy People 2020, a U.S. federal initiative that sets 10-year national health objectives, established targets of 90% pneumococcal vaccination coverage among adults aged ≥ 65 years and 60% among high-risk adults aged 19–64 years, but these benchmarks were not achieved [12]. By 2018, only about 69% of older adults and 24% of at-risk younger adults reported ever receiving a pneumococcal vaccination [13]. Healthy People 2030 continues to prioritize improving adult pneumococcal vaccination as a public health goal.

Although vaccination coverage and disparities have been examined previously, most national studies predate the COVID-19 pandemic and/or end before the 2022 ACIP shift to PCV15/PCV20. As a result, less is known about how pneumococcal vaccine uptake changed during the pandemic and whether patterns shifted in the early post-PCV20 era when recommendations were simplified. Contemporary, post-pandemic evidence is needed to guide strategies that reduce persistent gaps in adult coverage.

Therefore, this study examines trends over time and factors associated with pneumococcal vaccine uptake among adults in the United States from 2019 to 2024. By analyzing nationally representative data, we aim to [1] describe annual trends in pneumococcal vaccination coverage over the study period [2], identify demographic, socioeconomic, health-related, and healthcare access factors associated with pneumococcal vaccination uptake, including disparities across key subgroups (e.g., age group, race/ethnicity, and insurance status), and [3] provide evidence-based recommendations for targeted public health interventions to improve pneumococcal disease prevention strategies.

## Materials and methods

### Study design and data source

We conducted a pooled cross-sectional study using data from six recent cycles (2019–2024) of the National Health Interview Survey (NHIS). NHIS is an annual survey conducted by the CDC’s National Center for Health Statistics since 1957 to collect information on a broad range of health topics [14]. It employs a stratified, multistage sampling design to obtain data from the U.S. civilian noninstitutionalized population through household interviews. Data were collected primarily through computer-assisted personal interviewing, supplemented by telephone interviews. Detailed documentation, including questionnaires and data sets, is publicly available at https://www.cdc.gov/nchs/nhis/documentation/index.html. We selected 2019–2024 to provide the most recent pre-pandemic baseline (2019) and the subsequent pandemic and post-pandemic years available.

### Variables

The outcome variable was pneumococcal vaccine uptake, as reported by survey participants. NHIS collected this information using the question: “*A pneumonia shot is also known as a pneumococcal vaccine. Have you EVER had a pneumonia shot?*” Possible responses included “Yes”, “No”, “Refused”, or “Don’t know”.

The selection of independent variables was informed by prior literature on the topic, theoretical relevance and the variables consistently captured in the NHIS related to demographic, socioeconomic, healthcare access, and clinical characteristics. Respondents’ age was collected as a continuous variable in the survey, but we categorized it into four groups: 18–34, 35–54, 55–64, and 65 + years. This was done to reflect policy-relevant strata, particularly the 65 + years group for which the pneumococcal vaccination is universally recommended under ACIP guidelines, and to facilitate interpretation of vaccination patterns across the adult life course. Education level was recoded into three categories: below high school, high school graduate, and some college or more. The ratio of family income to poverty threshold was recorded as a value between 0 and 5 (top-coded). We recategorized values below 1 into “income below poverty line” and values above 1 into “income above poverty line” categories following a widely used and policy-relevant cutoff in public health research [15, 16]. Smoking status was assessed using the standard CDC measure of lifetime smoking, defined as having smoked at least 100 cigarettes in one’s lifetime (yes/no). Table 1 provides a detailed list of these variables along with their measurement levels.

**Table 1:** List of independent variables and their measurement

Independent variables	Level of measurement
Sex	Male, female
Age group (in years)	18–34, 35–54, 55–64, 65+
Race	Hispanic, non-Hispanic White, non-Hispanic Black, non-Hispanic Asian, Others
US citizen	Yes, no
Education	Below high school, high school graduate, some college or more
Ratio of family income to poverty threshold (PIR)	Income below poverty line, income above poverty line
Region	Midwest, Northeast, South, West
Residence	Large central metro, large fringe metro, medium and small metro, nonmetropolitan
Have a usual place for care	Yes, no
Delayed medical care due to cost	Yes, no
Insurance coverage	Covered, not covered
Time since last saw doctor	Within the past year, beyond the past year or never
Provide medical care to patients	Yes, no
Ever smoked 100 cigarettes	Yes, no
General health status	Poor, fair, good, very good, excellent
Ever had asthma	Yes, no
Ever had COPD	Yes, no
Flu vaccine (past 12 months)	Yes, no
Ever had cancer	Yes, no
Ever had diabetes	Yes, no

### Statistical analysis

Descriptive statistics were used to summarize the characteristics of the study population. Since all variables were analyzed in categorical form, results were presented as frequencies and weighted percentages with corresponding standard errors. Univariable logistic regression was used to estimate unadjusted associations between each covariate and pneumococcal vaccination uptake, and multivariable logistic regression was used to estimate adjusted associations. The overall model performance was assessed using the Nagelkerke pseudo-R^2^, which reflects the improvement of the fitted model over a null model, i.e. a model with no covariates. Model calibration and goodness of fit was assessed graphically by comparing observed and predicted probabilities with a 45-degree reference line alongside the Hosmer-Lemeshow test, although p-value was not of focus given the large sample size [17]. Results from regression analysis were presented as odds ratio (OR) with corresponding 95% confidence interval (CI). Variables with OR whose CI did not include 1.00 were considered to have statistically significant association with the outcome.

Given the relatively low proportion of missing data (less than 4% for any covariate) and no clear evidence of systematic missingness related to the outcome or covariates, a complete case analysis was performed. To analyze temporal changes in pneumococcal vaccine uptake, we calculated annual vaccination rates from 2019 to 2024 and visualized the trend using a line graph. It is to be noted that data collection in 2020 was affected by COVID-19 related disruptions (e.g., shift to telephone interviews and lower response rates). However, NHIS implemented weighting modifications to mitigate corresponding biases which enables comparability across cycles [18]. Several potential sources of bias were considered in the study design and analysis. Vaccination status was self-reported and may be subject to recall bias. To address confounding, we adjusted for a comprehensive set of demographic, socioeconomic, healthcare access, and clinical variables. Selection bias due to exclusion of respondents with missing data was mitigated using a large, pooled sample and application of survey weights. In addition, use of complex survey design features, including strata, clusters, and sampling weight information helped account for differential probabilities of selection and nonresponse. Statistical analyses were conducted using the R programming language. All statistical tests were two-sided, and a p-value of < 0.05 was considered statistically significant. This analysis adheres to the Preferred Reporting Items for Complex Sample Survey Analysis (PRICSSA) guidelines for secondary analyses of nationally representative survey data [19].

## Results

### Characteristics of the survey participants

A total of 182,849 respondents were initially identified across the six NHIS survey cycles (2019–2024). Participants with missing information on pneumococcal vaccination status (*n* = 8,040) were excluded. Additional exclusions were made for missing covariate data. The extent of missing data varied across covariates; the highest level of missingness was observed for the variable “US citizen” (*n* = 6,410). After these exclusions, the final analytic sample consisted of 166,948 eligible adults with complete data on all variables across the survey cycles.

Table 2 represents the characteristics of the survey participants. The sample was nearly equally distributed by gender (male 48.2% versus female 51.8%), while non-Hispanic White participants were the most represented (63.1%). A large proportion of participants were aged under 55 years, although nearly a quarter of participants were aged 65 years or older. Socioeconomically, the majority of respondents possessed at least some college education (61.6%), lived above the poverty line (90.1%), and held U.S. citizenship (91.9%). Participants were distributed across US regions, with the largest proportion residing in the South (38.4%), followed by the West, Midwest, and Northeast. Residential patterns were similarly balanced, with nearly equal proportions of adults living in large central metropolitan areas and medium or small metropolitan regions. Access to healthcare was generally high; approximately 90% of participants reported having health insurance and a usual place of care.

**Table 2:** Characteristics of the survey participants from NHIS 2019–2024 (*N* = 166,948)

Variable	Levels	Unweighted frequency	Weighted % ± SE
Sex	Male	75,758	48.2 ± 0.2
	Female	91,190	51.8 ± 0.2
Age group	18–34 years	33,586	28.4 ± 0.2
	35–54 years	49,525	32.3 ± 0.2
	55–64 years	30,210	16.7 ± 0.1
	65 + years	53,627	22.5 ± 0.2
Race	Hispanic	22,587	16.9 ± 0.6
	Non-Hispanic White	113,672	63.1 ± 0.7
	Non-Hispanic Black	17,465	11.6 ± 0.4
	Non-Hispanic Asian	8,948	5.8 ± 0.2
	Others	4,276	2.7 ± 0.2
US citizen	Yes	156,526	91.9 ± 0.2
	No	10,422	8.1 ± 0.2
Education	Below high school	14,080	10.8 ± 0.2
	High school graduate	42,266	27.6 ± 0.2
	Some college or more	110,602	61.6 ± 0.4
PIR	Income below poverty line	16,625	9.9 ± 0.2
	Income above poverty line	150,323	90.1 ± 0.2
Region	Midwest	37,502	21.0 ± 0.6
	Northeast	27,131	17.2 ± 0.6
	South	60,908	38.4 ± 0.9
	West	41,407	23.4 ± 0.8
Residence	Large central metro	48,261	30.4 ± 1.1
	Large fringe metro	38,242	24.8 ± 1.1
	Medium and small metro	52,095	30.5 ± 1.4
	Nonmetropolitan	28,350	14.3 ± 0.6
Have a usual place for care	Yes	152,728	90.0 ± 0.2
	No	14,220	10.0 ± 0.2
Delayed medical care due to cost	Yes	11,868	7.7 ± 0.1
	No	155,080	92.3 ± 0.1
Insurance coverage	Covered	154,238	90.1 ± 0.2
	Not covered	12,710	9.9 ± 0.2
Time since last saw doctor	Within the past year	143,657	84.0 ± 0.2
	Beyond the past year or never	23,291	16.0 ± 0.2
Provide medical care to patients	Yes	12,752	7.9 ± 0.1
	No	154,196	92.1 ± 0.1
Ever smoked 100 cigarettes	Yes	63,099	34.6 ± 0.3
	No	103,849	65.4 ± 0.3
General health status	Poor	5,960	3.2 ± 0.1
	Fair	19,726	11.1 ± 0.1
	Good	48,505	28.6 ± 0.2
	Very good	57,177	34.1 ± 0.2
	Excellent	35,580	22.9 ± 0.2
Ever had asthma	Yes	23,466	14.3 ± 0.1
	No	143,482	85.7 ± 0.1
Ever had COPD	Yes	9,569	4.6 ± 0.1
	No	157,379	95.4 ± 0.1
Flu vaccine (past 12 months)	Yes	86,025	46.9 ± 0.3
	No	80,923	53.1 ± 0.3
Ever had cancer	Yes	21,737	10 ± 0.1
	No	145,211	90 ± 0.1
Ever had diabetes	Yes	18,287	9.7 ± 0.1
	No	148,661	90.3 ± 0.1

These distributions were consistent with national estimates. The health insurance coverage rate (90.1%) aligned with national estimates of 90–92% from Census Bureau data [20]. The racial and ethnic composition also closely approximated 2020 Census figures for adults aged 18 years and older, with non-Hispanic White (63.1% vs. 61%), Hispanic (16.9% vs. 17%), and non-Hispanic Black (11.6% vs. 12%) adults well represented [21]. Educational attainment mirrored national patterns, with approximately 62% reporting some college or higher [22]. The proportion living below the poverty threshold (9.9%) was comparable to national rates of 10–11% during this period [23].

Regarding health behaviors and status, over one-third of participants (34.6%) reported a history of smoking at least 100 cigarettes, while nearly half (46.9%) had received a flu vaccine in the past year. Although the vast majority of adults rated their general health as good to excellent, chronic comorbidities were notable: asthma was the most prevalent condition (14.3%), followed by cancer and diabetes (approximately 10% each), and COPD (4.6%).

### Trend of pneumococcal vaccination uptake across the survey cycles

**Fig. 1:**
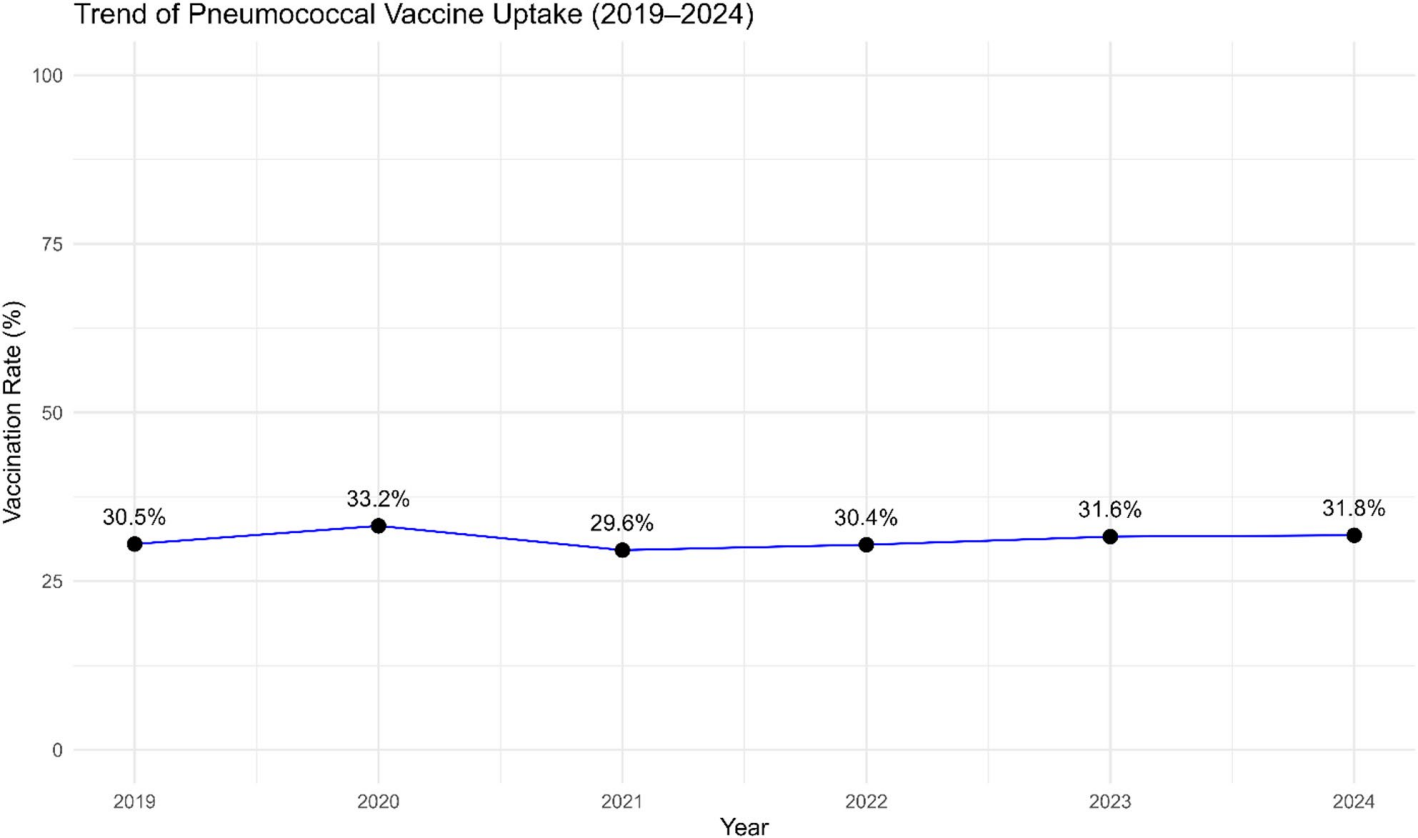
Annual pneumococcal vaccination uptake among U.S. adults, 2019–2024. Weighted percentages from pooled National Health Interview Survey data (*N* = 166,948)

Figure 1 shows annual pneumococcal vaccination coverage among adults from 2019 to 2024. Coverage fluctuated modestly, ranging from 29.6% in 2021 to 33.2% in 2020, but showed no meaningful increase over the six-year period. This stagnation is notable given that “ever vaccinated” is a cumulative measure, newly vaccinated individuals add to coverage each year while those previously vaccinated remain counted. Given natural population turnover, flat coverage suggests new uptake is only keeping pace with the loss of previously vaccinated individuals rather than exceeding it, leaving overall coverage stagnant.

### Weighted prevalence of pneumococcal vaccination across respondent characteristics

Table 3 shows substantial variation in pneumococcal vaccination uptake across demographic, socioeconomic, and healthcare-related characteristics. Vaccination prevalence was higher among females compared to males (26.7% vs. 22.8%) and increased markedly with age, ranging from 9.6% among adults aged 18–34 years to 65.7% among those aged ≥ 65 years. Coverage varied across racial groups with the lowest prevalence observed among Hispanic adults.

**Table 3:** Association between pneumococcal vaccination uptake and respondent characteristics

		Vaccinated	
Variables	Levels	Yes (*n*, Weighted %)	No (*n*, Weighted %)
Sex	Male	21,452 (22.8)	54,306 (77.2)
	Female	30,642 (26.7)	60,548 (73.3)
Age group	18–34 years	3,263 (9.6)	30,323 (90.4)
	35–54 years	5,449 (10.6)	44,076 (89.4)
	55–64 years	7,243 (23.0)	22,967 (77.0)
	65 + years	36,139 (65.7)	17,488 (34.3)
Race	Hispanic	3,733 (13.9)	18,854 (86.1)
	Non-Hispanic White	40,820 (29.1)	72,852 (70.9)
	Non-Hispanic Black	4,607 (20.9)	12,858 (79.1)
	Non-Hispanic Asian	1,822 (19.8)	7,126 (80.2)
	Others	1,112 (20.9)	3,164 (79.1)
US citizen	Yes	50,944 (26.1)	105,582 (73.9)
	No	1,150 (10.5)	9,272 (89.5)
Education	Below high school	4,250 (23.7)	9,830 (76.3)
	High school graduate	13,401 (24.6)	28,865 (75.4)
	Some college or more	34,443 (25.1)	76,159 (74.9)
PIR	Income below poverty line	4,736 (22.4)	11,889 (77.6)
	Income above poverty line	47,358 (25.1)	102,965 (74.9)
Region	Midwest	12,541 (26.8)	24,961 (73.2)
	Northeast	8,759 (25.5)	18,372 (74.5)
	South	18,456 (23.9)	42,452 (76.1)
	West	12,338 (24.0)	29,069 (76.0)
Residence	Large central metro	13,119 (22.0)	35,142 (78.0)
	Large fringe metro	11,947 (24.6)	26,295 (75.4)
	Medium and small metro	17,075 (26.0)	35,020 (74.0)
	Nonmetropolitan	9,953 (28.6)	18,397 (71.4)
Have a usual place for care	Yes	50,703 (26.6)	102,025 (73.4)
	No	1,391 (8.3)	12,829 (91.7)
Delayed medical care due to cost	Yes	2,417 (17.3)	9,451 (82.7)
	No	49,677 (25.4)	105,403 (74.6)
Insurance coverage	Covered	51,140 (26.7)	103,098 (73.3)
	Not covered	954 (7.2)	11,756 (92.8)
Time since last saw doctor	Within the past year	49,738 (27.9)	93,919 (72.1)
	Beyond the past year or never	2,356 (8.5)	20,935 (91.5)
Provide medical care to patients	Yes	2,914 (20.1)	9,838 (79.9)
	No	49,180 (25.2)	105,016 (74.8)
Ever smoked 100 cigarettes	Yes	23,696 (31.3)	39,403 (68.7)
	No	28,398 (21.4)	75,451 (78.6)
General health status	Poor	3,175 (48.5)	2,785 (51.5)
	Fair	8,891 (38.9)	10,835 (61.1)
	Good	16,994 (28.0)	31,511 (72.0)
	Very good	16,082 (22.0)	41,095 (78.0)
	Excellent	6,952 (15.0)	28,628 (85.0)
Ever had asthma	Yes	9,199 (32.0)	14,267 (68.0)
	No	42,895 (23.6)	100,587 (76.4)
Ever had COPD	Yes	6,244 (60.4)	3,325 (39.5)
	No	45,850 (23.1)	111,529 (76.9)
Flu vaccine (past 12 months)	Yes	40,993 (40.1)	45,032 (59.9)
	No	11,101 (11.3)	69,822 (88.7)
Ever had cancer	Yes	12,831 (53.7)	8,906 (46.2)
	No	39,263 (21.6)	105,948 (78.4)
Ever had diabetes	Yes	10,536 (51.8)	7,751 (48.2)
	No	41,558 (21.9)	107,103 (78.1)

Socioeconomic and access-related differences were also evident. U.S. citizens reported higher vaccination prevalence compared to non-U.S. citizens (26.1% vs. 10.5%). Individuals with a usual place of care (26.6%), not facing cost-related delays in medical care (25.4%), with insurance coverage (26.7%), and those who had a contact with a doctor within the past year (27.9%) had substantially higher vaccination prevalence compared to those without such access.

Individuals reporting a poor health status (48.5%), chronic conditions, including asthma (32.0%), COPD (60.4%), cancer (53.7%), and diabetes (51.8%), had markedly higher vaccination coverage compared to those without these conditions. Receipt of influenza vaccination was also strongly associated with higher pneumococcal vaccination prevalence, with coverage substantially higher among those who received a flu vaccine (40.1%) compared to those who did not (11.3%).

## Factors associated with pneumococcal vaccination uptake

Table 4 presents the results of both univariable and multivariable logistic regression models examining factors associated with pneumococcal vaccination uptake. In unadjusted analysis with univariable models, female sex, older age, non-Hispanic Whites, U.S. citizenship, higher educational attainment, and having greater healthcare access were all associated with higher odds of receiving pneumococcal vaccination. Similarly, the presence of chronic conditions and receipt of influenza vaccination were associated with higher uptake.

**Table 4:** Univariable and multivariable logistic regression analysis of factors associated with pneumococcal vaccination uptake among U.S. adults, NHIS 2019–2024 (*N* = 166,948)

		Univariable model results		Multivariable model results	
		Unadjusted odds ratio	95% CI	Adjusted odds ratio	95% CI
Sex	Male	0.80	0.79, 0.83	0.91	0.88, 0.94
	Female (Ref.)				
Age group	18–34 years (Ref.)				
	35–54 years	1.11	1.05, 1.18	0.86	0.81, 0.92
	55–64 years	2.80	2.64, 2.98	1.61	1.51, 1.72
	65 + years	18.04	17.13, 19.00	9.39	8.84, 9.96
Race	Hispanic (Ref.)				
	Non-Hispanic White	2.54	2.41, 2.67	1.44	1.35, 1.54
	Non-Hispanic Black	1.63	1.52, 1.75	1.25	1.15, 1.36
	Non-Hispanic Asian	1.53	1.40, 1.67	1.12	1, 1.25
	Others	1.64	1.48, 1.81	1.41	1.22, 1.63
US citizen	Yes (Ref.)				
	No	0.33	0.31, 0.36	0.88	0.79, 0.97
Education	Below high school (Ref.)				
	High school graduate	1.05	0.99, 1.11	1.29	1.2, 1.38
	Some college or more	1.08	1.02, 1.14	1.40	1.30, 1.50
PIR	Income below poverty line (Ref.)				
	Income above poverty line	1.16	1.10, 1.22	0.94	0.88, 1.01
Region	Midwest (Ref.)				
	Northeast	0.93	0.88, 0.99	0.89	0.83, 0.94
	South	0.86	0.81, 0.90	0.91	0.87, 0.96
	West	0.86	0.82, 0.91	1.03	0.97, 1.09
Residence	Large central metro (Ref.)				
	Large fringe metro	1.16	1.10, 1.21	1.02	0.97, 1.07
	Medium and small metro	1.25	1.18, 1.32	1.02	0.97, 1.08
	Nonmetropolitan	1.42	1.34, 1.51		0.94, 1.07
Have a usual place for care	Yes (Ref.)				
	No	0.25	0.23, 0.27	0.81	0.74, 0.88
Delayed medical care due to cost	Yes (Ref.)				
	No	1.63	1.54, 1.73	0.97	0.9, 1.04
Insurance coverage	Covered (Ref.)				
	Not covered	0.21	0.20, 0.23	0.89	0.81, 0.98
Time since last saw doctor	Within the past year	4.16	3.92, 4.41	1.41	1.31, 1.51
	Beyond the past year or never (Ref.)				
Provide medical care to patients	Yes (Ref.)				
	No	1.34	1.27, 1.41	1.03	0.96, 1.1
Ever smoked 100 cigarettes	Yes (Ref.)				
	No	0.60	0.58, 0.61	0.84	0.81, 0.87
General health status	Poor (Ref.)				
	Fair	0.68	0.63, 0.72	0.97	0.89, 1.06
	Good	0.41	0.39, 0.44	0.76	0.7, 0.83
	Very good	0.3	0.28, 0.32	0.66	0.61, 0.72
	Excellent	0.19	0.17, 0.20	0.56	0.51, 0.61
Ever had asthma	Yes	1.52	1.46, 1.58	1.72	1.64, 1.8
	No (Ref.)				
Ever had COPD	Yes	5.10	4.81, 5.40	2.04	1.9, 2.2
	No (Ref.)				
Flu vaccine (past 12 months)	Yes (Ref.)				
	No	0.19	0.18, 0.20	0.29	0.28, 0.30
Ever had cancer	Yes	4.23	4.07, 4.39	1.36	1.3, 1.42
	No (Ref.)				
Ever had diabetes	Yes	3.82	3.67, 3.98	1.84	1.74, 1.95
	No (Ref.)				

In the multivariable model, several factors remained independently associated with vaccination uptake. Adults aged ≥ 65 years were found to have a substantially higher odds of pneumococcal vaccination compared with adults aged 18–34 years (OR = 9.39; 95% CI: 8.84–9.96). Race and ethnicity remained significant; adjusting for socioeconomic factors, non-Hispanic White and Black adults had higher odds of vaccination relative to Hispanic adults (OR = 1.44; 95% CI: 1.35–1.54) and (OR = 1.25; 95% CI: 1.15–1.36) respectively. Beyond racial and socioeconomic factors, citizenship status emerged as an independent predictor of uptake. Non-U.S. citizens had 12% lower odds of being vaccinated compared to citizens (OR = 0.88; 95% CI: 0.79–0.97). Higher educational attainment was positively associated with uptake with clear educational gradient: adults with high school education and those with some college or more compared had a higher odds compared to adults with less than a high school education (OR = 1.29; 95% CI: 1.20–1.38) and (OR = 1.40; 95% CI: 1.30–1.50) respectively.

Healthcare access and utilization continued to drive uptake in the adjusted model. Having a recent doctor visit significantly increased the odds of vaccination, whereas lacking insurance (OR = 0.89; 95% CI: 0.81–0.98) or a usual place of care (OR = 0.81; 95% CI: 0.74–0.88) significantly reduced them. Chronic comorbidities, specifically COPD, diabetes, asthma, and cancer, remained independent predictors of higher vaccine uptake. Finally, the strongest behavioral predictor was receipt of the influenza vaccine; individuals who had not received a flu shot in the past year had substantially lower odds of receiving the pneumococcal vaccine compared to those who had (OR = 0.29; 95% CI: 0.28–0.30). Overall, these findings highlight the central role of age, healthcare access, and preventive health behaviors in shaping pneumococcal vaccination uptake.

**Fig. 2:**
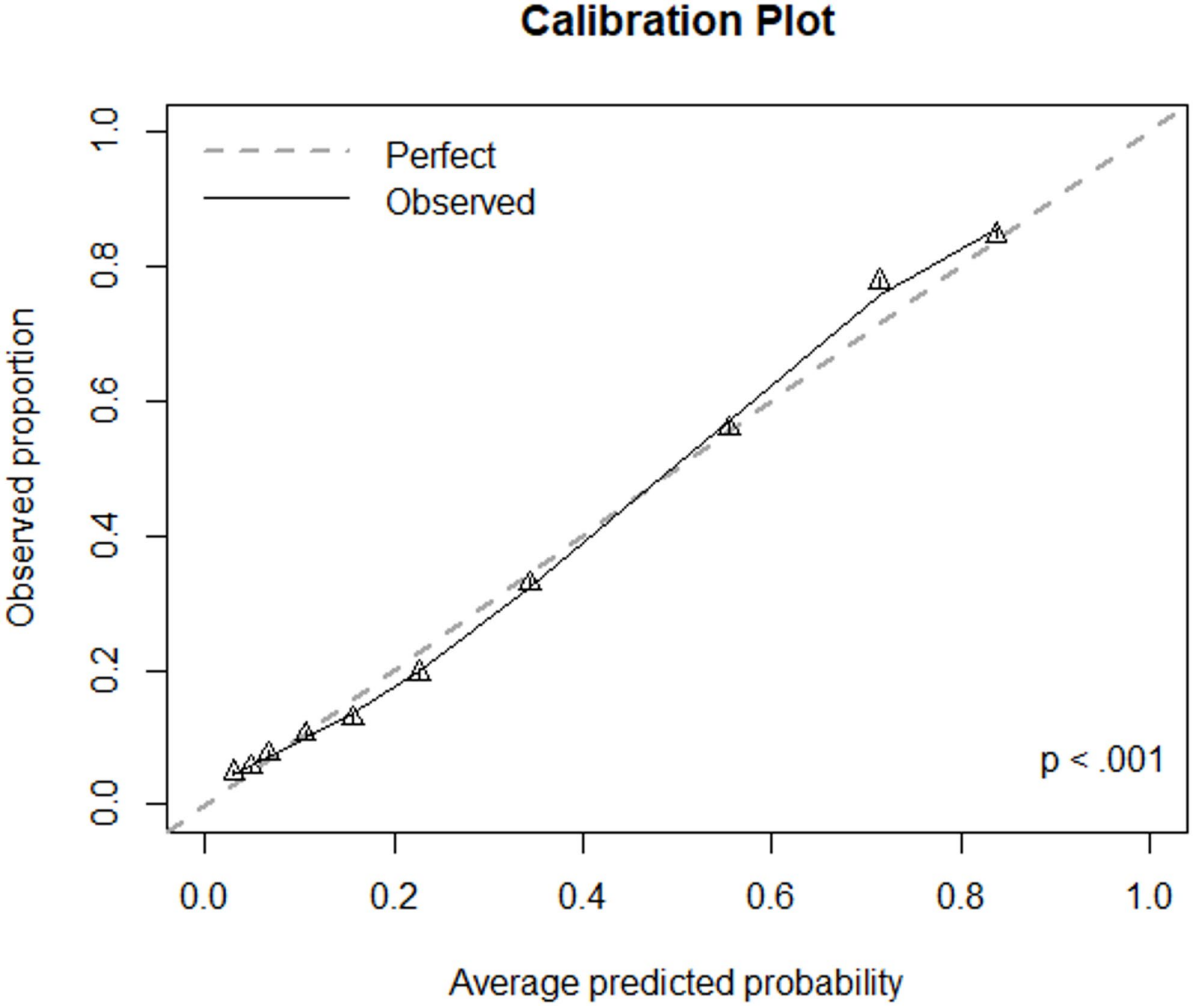
Calibration plot for the multivariable logistic regression model, including Hosmer-Lemeshow test p-value

The multivariable model demonstrated a strong overall fit, with a Nagelkerke pseudo-R^2^ of 0.43. The calibration plot demonstrated good agreement between observed and predicted probabilities, with majority of the points lay close to the 45-degree reference line, see Fig. 2. Although the Hosmer-Lemeshow test was statistically significant (*p* < 0.001), this likely reflects the large sample size, where even small deviations can yield highly significant results.

## Discussion

In this large, nationally representative sample of U.S. adults, pneumococcal vaccination coverage remained low and showed minimal improvement across the six survey cycles. Uptake was concentrated among older adults and those living with chronic health conditions, whereas younger adults were vaccinated far less often despite longstanding recommendations to do so. Patterns in our data also point to the importance of consistent engagement with healthcare, individuals who had recently seen a clinician or received an influenza vaccine were substantially more likely to be vaccinated. However, broad disparities persisted across racial and educational groups and among adults without stable access to care, indicating that opportunities for pneumococcal vaccination continue to be unevenly distributed.

### Overall trend and uptake of vaccination

Between 2019 and 2024, overall pneumococcal vaccination coverage among U.S adults remained stagnant, with rates fluctuating marginally from 33.2% to 29.6%. These figures remain well below the Healthy People 2020 target of 60%, continuing a documented decade-long trend of inadequate improvement reported by CDC and recent studies. This persistent stagnation occurred against a backdrop of significant policy evolution, including updated ACIP guidelines in 2019 and subsequent recommendations for new conjugate vaccines (PCV15 and PCV20) [11].

The temporal pattern of coverage reveals a potential pandemic-related disruption. A modest peak in 2020 likely reflects a transient rise in health awareness during the initial phase of the COVID-19 crisis [24, 25]. However, this gain was not sustained in 2021, a decline that coincides with the peak pandemic period. This may be suggestive of disruptions of routine preventive care, consistent with the CDC’s own analysis which identified a clear “pandemic effect” wherein adults who became eligible for vaccination during 2020–2021 had significantly lower uptake, potentially related to deferred healthcare visits [26, 27]. Beyond acute care disruptions, public ambivalence toward adult vaccination may have also played a role. Although not directly measured in NHIS, vaccine hesitancy has been reported to have intensified in some groups during the pandemic, reinforcing pre-existing skepticism toward vaccination [28, 29]. This dynamic, compounded by care disruptions, may have limited improvements in pneumococcal immunization.

However, because our outcome is cumulative (“ever vaccinated”), our data cannot directly detect short-term changes in incident vaccination, individuals vaccinated years ago remain in the “vaccinated” category regardless of current-year uptake. An alternative explanation for the observed stagnation is accelerated population turnover during the pandemic. COVID-19 disproportionately affected older adults, who also had the highest vaccination rates [30]; their loss from the population may have offset gains from newly vaccinated individuals. Under this scenario, new vaccination would need to exceed replacement simply to maintain coverage levels. Without age-specific or newly-eligible subgroup analyses, these interpretations remain speculative.

Suboptimal adult pneumococcal vaccination is not unique to the United States. In Europe, average coverage is approximately 24% among older adults and 18% among clinical risk groups, with substantial variation across countries ranging from 1% to 70% [31]. A recent report highlighted that despite recommendations and reimbursement policies in several European countries, adult pneumococcal vaccination rates remain low [31]. This variability reflects differences in national guidelines, vaccine type recommendations, and implementation strategies across countries [31, 32]. These patterns suggest that challenges in achieving adequate adult pneumococcal vaccination coverage extend beyond any single healthcare system.

### Age as the strongest predictor

Importantly, age emerged as the strongest correlate of vaccination status, though this partly reflects the structure of recommendations. Pneumococcal vaccination is universally recommended for adults ≥ 65 years, whereas younger adults, who served as the reference category (18–34 years), are only selectively targeted based on risk conditions. This structurally inflates the comparison, partly explaining the nearly 9.4 times higher odds of vaccination among adults ≥ 65 years, of whom 65.7% reported vaccination. Nevertheless, while the reference group may inflate the odds ratio, it does not explain the actual coverage level 65.7% still falls far below the Healthy People 2020 goal of 90% for this age group [12, 27]. The higher uptake is also consistent with the fact that pneumococcal vaccination has been routinely recommended for this age group since the 1980s, meaning older adults have had decades to accumulate vaccination and that they have more frequent healthcare encounters where vaccination can be offered. Prior to the pandemic, coverage in this group had plateaued in the mid-60% range and never approached target levels [27]. The persistent shortfall below the Healthy People goal underscores longstanding challenges in vaccinating older adults, even as newer conjugate vaccines became available.

While uptake in the ≥ 65 age group remains suboptimal, it is substantially lower among younger adults. In our analysis, only 23.0% of adults aged 55–64 years had ever received a pneumococcal vaccine, a finding consistent with earlier studies reporting persistently low coverage among high-risk younger adults and highlighting a longstanding gap in risk-based immunization [33, 34]. Pneumococcal vaccination among adults aged 19–64 years with underlying conditions has similarly remained low in prior research, with uptake ranging from 6% after one year to 21% after five years of follow-up [35], suggesting that early opportunities for prevention are often missed, which is particularly concerning because many chronic conditions that elevate pneumococcal disease risk, including COPD, diabetes, asthma, and cancer, commonly emerge well before age 65 [10, 36].

### Race and ethnicity: disparities that persist despite adjustment

We observed that Black and Hispanic adults have significantly lower pneumococcal vaccination rates than White adults. Our findings are consistent with those documented for other adult vaccines, including seasonal influenza and showed a similar pattern as observed by the CDC’s 2022 survey that showed 69% of White seniors had been vaccinated, versus only 54% of Black and 42% of Hispanic [27]. Hispanic adults had the lowest vaccination coverage of less than half the rate for non-Hispanic White adults. This disparity narrowed after we adjusted for income, education, and insurance, but it didn’t disappear. Non-Hispanic Black adults showed 9% lower uptake than White adults, though notably, adjustment for socioeconomic factors attenuated the disparity for Black adults, suggesting that much of the Black, White gap may be partly explained by income, education, and insurance differences. In contrast, the Hispanic-White disparity persisted even after adjustment, indicating that socioeconomic factors explain part, but not all of the lower coverage among Hispanic adults.

Several mechanisms documented in prior research may help explain the persistent Hispanic-White disparity observed even after socioeconomic adjustment. First, limited English proficiency which has been shown to reduce preventive service use by impeding communication, understanding of vaccine indications, and navigation of the healthcare system, can lowers vaccination uptake [37, 38]. Similarly, the absence of culturally tailored communication may reduce relevance and credibility of vaccine messaging, particularly for first-generation or less acculturated adults, thereby weakening intent to vaccinate [37, 39]. Differential access to healthcare settings where adult vaccines are routinely administered, such as primary-care clinics, pharmacies, and federally qualified health centers also contributes to disparities [37, 40].

Another plausible mechanism is mistrust in medical system. Large national and regional studies in the U.S and UK demonstrate that higher levels of medical mistrust are strongly associated with lower odds of receiving vaccines. For example, in one study, each additional point on a validated medical mistrust scale was linked to a 16% decrease in the odds of COVID-19 vaccination in a U.S national sample. Hispanic have a high rate of medical with recent studies placing it at 68.9%, meaning that nearly seven out of ten Hispanic adults report some level of mistrust in the medical system [41].

### Socioeconomic and access barriers

Our findings demonstrate that pneumococcal vaccination is shaped less by socioeconomic resources per se than by how those resources facilitate healthcare system engagement. The attenuation of income effects after adjustment suggests that income differences may operate through differences in insurance status, continuity of care, and provider contact, factors that may shape opportunities for vaccination. This aligns with evidence that adult immunization depends fundamentally on healthcare system touchpoints, and that individuals with infrequent clinical contact are far less likely to receive preventive services [42, 43]. The strong associations observed for insurance, usual source of care, and recent healthcare visits suggests that differential healthcare access is associated with differences in coverage.

Education operated differently. Its association with vaccination persisted after adjusting for access-related factors, indicating that education’s influence on vaccination is not solely mediated through income, insurance, or access, but reflects a different mechanism. This is consistent with prior research that showed that education attainment enhances the ability to interpret health information, increases receptivity to preventive care recommendations, and is associated with greater continuity of care [44]. These mechanisms may enable adults with higher educational attainment to capitalize more effectively on healthcare encounters and to initiate preventive care without strong provider prompting [44, 45]. Thus, education functions as an independent determinant of healthcare engagement and vaccination decision-making, not merely as a socioeconomic marker.

The persistence of low overall coverage despite widespread access to usual sources of care reveals a critical implementation gap. Though 90% of adults reported a regular care site, this access did not translate into adequate vaccination uptake, indicating that the bottleneck lies not only in reaching the healthcare system but in what occurs within it. The gap identified in several adult vaccine studies highlights that preventive care often remains opportunistic rather than systematic, resulting in missed opportunities even for those fully integrated into healthcare [46]. While expanding insurance and primary care access is crucial, it must be complemented by systematic vaccine eligibility assessments, clinical workflow modifications, and proactive vaccination strategies to achieve significant improvements in pneumococcal vaccine coverage.

### Health status and chronic conditions

The strong association between influenza vaccination and pneumococcal vaccination reflects the well-documented clustering of preventive health behaviors, but it also shows an important pattern in how adults engage with the healthcare system. Individuals who receive the influenza vaccine are, in general, those who maintain regular clinical contact, respond to preventive care recommendations, and view vaccination as a routine component of health maintenance. The link between the two vaccines may reflect a combination of behavioral dispositions, such as a general orientation toward preventive care, as well as structural factors, including more frequent opportunities for providers to offer or recommend additional vaccines during routine visits. In this context, the association is not only an indicator of co-acceptance but a marker of broader healthcare engagement and access that shape adult immunization. This finding points to opportunities for co-administration or at least concurrent promotion of both vaccines during clinical encounters, particularly during annual flu vaccination campaigns.

Notably, adults who had ever smoked 100 cigarettes showed higher vaccination rates than non-smokers (31.3% vs. 21.4%), with non-smokers having 16% lower adjusted odds. This may reflect, at least in part, consistency with risk-based recommendations, as smoking is an established indication for pneumococcal vaccination. However, given that only about one-third of smokers were vaccinated despite their elevated risk, this shows that risk-based recommendations are not consistently implemented. Weg et al. [47] showed that providers often underrecognize smoking as an independent indication preventive services, and most preventive services for smokers tend to focus narrowly on cessation counseling rather than broader preventive care.

### Strength and limitations

To our knowledge, few national analyses have evaluated pneumococcal vaccination trends among U.S. adults across the full 2019–2024 period. Most existing studies either end before the COVID-19 pandemic or prior to the 2022 ACIP updates and recent investigations assessing uptake after the new guidelines typically examine only the early post-recommendation period or focus on specific subpopulations [13, 35, 48, 49]. Using a Social-Ecological Model framework, our analysis integrates multilevel factors and extensive stratification across demographic, clinical, and geographic groups to identify subpopulations with persistently low coverage.

The use of a nationally representative dataset and validated survey measures enhances generalizability to community-dwelling adults, and pooling multiple NHIS waves increases precision and allows assessment of pandemic-related disruptions and policy shifts. Weighted sample characteristics closely approximated U.S. Census benchmarks for insurance coverage, racial/ethnic composition, educational attainment, and poverty rates, supporting the generalizability of our findings. The multivariable model demonstrated good overall fit (Nagelkerke pseudo-R² = 0.43) with adequate calibration, supporting the reliability of the estimated associations. Taken together, these features strengthen the interpretability and generalizability of the findings.

However, several limitations should be acknowledged. First, vaccination status was self-reported and, therefore, subject to recall errors, particularly in older adults. Second, the cross-sectional nature of the NHIS precludes causal inference, and unmeasured confounding may persist despite adjustment for a broad set of demographics, socioeconomic, clinical, and healthcare access factors. Third, NHIS samples only the non-institutionalized population and therefore excludes adults in long-term care, among whom pneumococcal disease risk is elevated. In addition, declining response rates during the COVID-19 pandemic may have introduced nonresponse bias, although NHIS weighting procedures were modified to mitigate known sources of such bias. The use of a complete case analytic approach may also introduce selection bias if missingness pattern is not completely at random, potentially affecting both internal validity and generalizability of the findings. However, in our study, the proportion of missing data was relatively small, and the use of survey weights helps mitigate potential bias related to sampling and nonresponse [50]. Finally, factors such as provider recommendation, vaccine hesitancy, medical mistrust, and clinical contraindications are not consistently captured in NHIS and may influence uptake. These limitations should be considered when interpreting the magnitude of estimates and residual disparities. Also, where we discuss these factors as potential explanations for observed patterns, they should be interpreted as hypotheses informed by prior literature rather than empirically measured mechanisms in our data.

## Conclusion

This study provides a comprehensive assessment of pneumococcal vaccination trends among U.S. adults from 2019 to 2024. Despite effective vaccines and national recommendations, substantial gaps in coverage persist, particularly among adults under 65 with risk conditions and racial and ethnic minorities. Vaccine uptake has shown minimal improvement over the study period, with notable declines among older adults during the COVID-19 pandemic, highlighting the fragility of preventive services during public health crises. By examining vaccination, we identified critical disparities across racial, socioeconomic, and healthcare access dimensions that require targeted intervention. These findings carry particular urgency given population aging and ongoing respiratory infection threats. The patterns we document can inform strategies to strengthen vaccine delivery systems, enhance provider-patient interactions, and address structural barriers to care. Future research should both examine behavioral and psychosocial factors not captured in NHIS, such as vaccine hesitancy, risk perception, and trust in healthcare and evaluate targeted interventions (e.g., provider prompts, standing orders, community-tailored outreach, pharmacy-based delivery) to inform strategies to improve uptake.

## Data Availability

The datasets analyzed during the current study are publicly available from the U.S. Centers for Disease Control and Prevention (CDC) National Center for Health Statistics (NCHS) National Health Interview Survey (NHIS). NHIS questionnaires, datasets, and documentation can be accessed through the NHIS documentation portal.

## Abbreviations

ACIP: Advisory committee on immunization practices
AOR: Adjusted odds ratio
CDC: Centers for disease control and prevention
CI: Confidence interval
COPD: Chronic obstructive pulmonary disease
NHIS: National health interview survey
PCV13: 13-valent pneumococcal conjugate vaccine
PCV15: 15-valent pneumococcal conjugate vaccine
PCV20: 20-valent pneumococcal conjugate vaccine
PPSV23: 23-valent pneumococcal polysaccharide vaccine
PIR: Poverty income ratio
